# 

**Published:** 1821-12-01

**Authors:** 


### Contents

OF

### No. 7, Vol. II. for DECEMBER, 1821.

Art. I.

Dr. Golis's Treatise on the Hydrocephalus Acutus; translated by Dr. Gooch - - - - - - - - - - - - - - - - 465

II.

Dr. Jackson on the Epidemic Yellow Fever of Cadiz and the South Coasts of Spain - - - - - - - - - - - - 485

III.

Mr. Wallace on Sulphureous Fumigations in Rheumatism, and Diseases of the Skin - - - - - - - - - - - - 507

IV.

Transactions of the Association of the Fellows and Licentiates of the King's and Queen's College of Physicians, Vol. III. [Continued from No. 6. p. 337.] - - - - -

Art. 6. Mr. Carmichael's Case of Amputation at the Hip-joint - - - - - - - - - - - - - - - 511

Case related by the Editors - - - - - - - - 514

—— 7. Cases of Tracheotomy, with Remarks - - - 516

Case of Abscess behind the Œsophagus - - - 518

—— 8. Dr. Grattan's Case of Gangrene from Mercury 521

—— 9. Dr. Brooke's Case of Liver Cough - - - - - 524

——10. Dr. Nicholl's Case of Erethism of the Brain - 529

——11. Dr. Nicholl's Case of Melena - - - - - - - 529

——12. Dr. M'Keever's Case of Ruptured Vagina - - 530

——13. Dr. Folloon's Case of Disease of the Heart - 532

——14. Dr. Lendrick's Case of Poisoning - - - - - 534

V.

PORRIGO, OR TINEA CAPITIS.

1. Mr. Plumbe on Ringworm of the Scalp - - - - - }
2. Dr. Alibert on Tinea Capitis - - - - - - - - - - } 534

VI.

Mr. Shaw's Manual for the Students of Anatomy - - - 551

VII.

1. Mr. Tully on the Plague of Malta, Gozo, Corfu, Cephalonia, &c. &c. - - - - - - - - - - - - - - - }

2. Dr. Hancock's Researches into the Laws and Phenomena of Pestilence, &c. - - - - - - - - - - - - - - } 569

☞ *See also, p.* 665.

VIII.

Dr. Ferguson on the Nature and History of Marsh Poison - 585

### IX.

SYPHILIS, PSEUDO-SYPHILIS, MERCURY.

1. Dr. Sintelaer's Scourge of Venus and Mercury
2. Mr. Bacot's Observations on Syphilis
3. Dr. Thompson on Mercury
4. Dr. Swediaur on Venereal Diseases
5. Mr. Harrison's Engravings, &c. - - - 597

### X.

A Manual of the Diseases of the Human Eye. By Dr. C. H. Weller; translated from the German, by G. C. Monteath, M.D. - - - 626

### XI.

Supplemental Review; or Quarterly Periscope of Practical Medicine, Surgery, &c.

Art. 1. Contusions on the Epigastrium - - - 662
2. Dr. Hancock on Pestilence - - - 665
3. Dr. Newman's Case of Internal hæmorrhage - - - 668
4. Dr. Wilson on Tincture of Poppy - - - 669
5. Sir Gilbert Blane on Hydrocephalus - - - 669
6. On Kinine, Cinchonine, and Sulphate of Kinine - 670
7 Dr. Platt on Epistaxis - - - 673
8. Dr. M'Leod on Prussic Acid - - - 673
9. Baron Larrey on Retinal Paralysis - - - 674
10. Dr. Meigs on Inverted Toe-nail - - - 675
11. Dr. Majendie on Extract of Opium - - - 675
12. Sementini on Nitrate of Silver - - - 676
13. Dr. Pinel, Junr. on Arachnitis - - - 677
14. Dr. Ulrich on Carditis - - - 679
15. M. Maunoir on Sarcocele - - - 682
16. Mr. Wheeler's Case of Dislocated Patella - - - 682
17. Dr. Sprengel's Case of Arteritis - - - 683
18. M. Montain on Ruptured Perineum - - - 684
19. Prosper Alpinus on Congestive Fever - - - 685

### XII.

Extra Limites.

Dr. Reeder's Remonstrance - - - 686

### XIII.

Bibliographical Record - - - 687

### XIV.

Miscellanies, Intelligence, Correspondence, &c. - - - 690

List of new Subscribers - - - 692

Medical Advertiser - - - 694

